# Beyond physical fitness: Pre-to-post hematological and physical fitness changes during an eight-week multi-component exercise program in sedentary adults aged 30–45 years

**DOI:** 10.1371/journal.pone.0354672

**Published:** 2026-07-28

**Authors:** Yasin Demircan, Vedat Ayan, Selami Yüksek, Fatih Gür, Burak Kural, Halit Şar

**Affiliations:** 1 Faculty of Sport Sciences, Trabzon University, Trabzon, Turkey; 2 Faculty of Sport Sciences, Pamukkale University, Denizli, Turkey; 3 Faculty of Sport Sciences, Sinop University, Sinop, Turkey; Versiti Blood Research Institute, UNITED STATES OF AMERICA

## Abstract

This exploratory study aimed to examine pre-to-post changes in physical fitness and selected hematological parameters during an 8-week multi-component exercise program in sedentary adults. The study used a single-group pre-test/post-test quasi-experimental design to describe temporal changes within the same participants. Thirty-six sedentary volunteers (15 females and 21 males) aged 30–45 years completed a multi-component program including cardiorespiratory endurance, strength, balance, and flexibility exercises for 45–50 minutes per session, 3 days per week, for 8 weeks. Paired-samples t-tests were used to compare pre-test and post-test measurements. Body weight and body mass index decreased, whereas estimated VO_2_max, vertical jump, flexibility, speed, agility, and balance performance showed statistically significant pre-to-post changes (all p < 0.001). Significant increases were also observed in hemoglobin, hematocrit, erythrocyte, leukocyte, and platelet values (all p < 0.001), whereas MCV and MCH did not change significantly (p > 0.05). Participation in the program coincided with favorable changes in body composition and physical performance and with measurable changes in selected hematological parameters. However, the absence of a control group and the lack of direct assessment of plasma volume, hydration status, erythropoietin, iron status, and hemoglobin mass preclude causal and mechanistic conclusions. The hematological findings may partly reflect hemoconcentration, plasma-volume shifts, or other physiological variation and should therefore be interpreted cautiously.

## Introduction

Digitalization, technological advancements, lifestyle changes driven by urbanization, and the comfort zones offered by modern life have reduced the necessity for individuals to be physically active, thereby facilitating the prevalence of a sedentary lifestyle. Today, physical inactivity is identified by the World Health Organization (WHO) as the fourth leading cause of death globally [1]. Furthermore, according to WHO data, 27.5% of adults worldwide engage in insufficient levels of physical activity [1]. While this rate rises to 31% in Europe, data from the Turkish Statistical Institute indicate that the majority of individuals aged 15 years and older in Türkiye reported no health-enhancing aerobic physical activity in 2022 [2].

Individuals who have low energy expenditure in daily life, spend prolonged periods sitting, and do not perform at least 150 minutes of moderate-intensity exercise per week are defined as “sedentary” [3]. This lifestyle is a major risk factor for the development of numerous chronic health problems, particularly cardiovascular diseases, obesity, type 2 diabetes, and musculoskeletal disorders. In this context, the development of exercise-based intervention programs aimed at reducing health risks in sedentary individuals and the scientific investigation of their effectiveness are of paramount importance.

Regular exercise has been associated with changes in physical fitness and selected biological parameters in sedentary adults. Previous studies have reported improvements in estimated or directly measured VO_2_max, muscular performance, and functional capacity following structured exercise programs, although the magnitude of change varies according to participant characteristics and intervention design [4]. Exercise may also be accompanied by changes in circulating hematological parameters [5,6]. However, variations in HGB, HCT, and RBC concentrations can be influenced by plasma volume, hydration status, iron availability, and other factors; therefore, changes in these measures do not necessarily indicate increased erythropoiesis or enhanced oxygen-carrying capacity.

However, the manifestation of these effects may vary depending on the type and duration of the exercise program applied, as well as individual characteristics. Recent studies demonstrate that 12-week regular exercise programs yield significant improvements in both cardiorespiratory fitness and metabolic health in sedentary individuals. For instance, a randomized controlled trial conducted by Navarro-Lomas et al. [7] reported that a 12-week structured exercise intervention enhanced physical fitness in sedentary middle-aged individuals. Similarly, another study examined the effects of different exercise types on steroid hormones in physically inactive middle-aged adults and observed positive results [8]. Furthermore, individualized aerobic exercise programs were found to increase cardiorespiratory fitness and general physical activity levels, particularly in middle-aged and older individuals with multimorbidity [9].

These findings suggest that structured exercise programs may support physical fitness and metabolic health in sedentary adults [9]. However, studies that evaluate physical fitness and hematological variables concurrently in sedentary adults aged 30–45 years remain limited. Examining these outcomes concurrently may provide a broader descriptive picture of the changes observed during a multi-component exercise period. Nevertheless, because circulating blood-cell concentrations can be affected by hydration and plasma-volume shifts, hematological findings require cautious interpretation.

In this context, the present exploratory study aimed to examine pre-to-post changes in physical fitness and selected hematological parameters following an 8-week multi-component exercise program in sedentary adults aged 30–45 years. Given the single-group pre-test/post-test quasi-experimental design, the study was not intended to establish causal effects but rather to provide preliminary observations regarding temporal changes in body composition, estimated aerobic capacity, motor performance, and hematological markers within this demographic. It was hypothesized that participation in the structured multi-component exercise program would be associated with favorable changes in physical fitness outcomes and selected hematological parameters.

## Materials and methods

### Research design

This study used a single-group pre-test/post-test quasi-experimental design. The design permits the examination of within-participant changes between two time points; however, without a control group, the observed changes cannot be attributed exclusively to the exercise program [10].

### Participants

The study group was determined using the “criterion sampling” strategy. A total of 36 volunteers (15 females, 21 males) aged between 30 and 45 years were included in the study. A power analysis was conducted to evaluate the adequacy of this sample size. The results indicated that a sample size of 36 participants provides a statistical power of 0.83 to detect a medium effect size (Cohen's d = 0.50) with an alpha level of 0.05. The primary inclusion criteria were having a “sedentary” lifestyle and no history of chronic diseases. Sedentary status was defined as not having participated in moderate or vigorous-intensity regular physical activity within the last six months [11]. Participants were recruited for this study between 20 January 2023 and 20 March 2023. Written informed consent was obtained from all participants. The study was approved by the Trabzon University Social and Humanities Research and Publication Ethics Committee (Decision No: 2300005310).

### Exercise program

The exercise protocol was planned for 8 weeks, 3 days a week. Each session consisted of warm-up (5–10 min), main exercise phase (25–30 min), and cool-down (5–10 min) stages. The main exercise phase included: rope skipping, Y-T-W exercises, medicine ball throw, T-push-up, shoulder press (airplane movement), hip bridge, plank, sit-ups, plank with forward reach, lunges, squat jumps, drop squats, and airplane exercise. The protocol was organized as a progressive multi-component program in accordance with established exercise-prescription principles [12]. To ensure the reproducibility of the intervention, the detailed progression of exercise volume, intensity (%HRmax) and training modalities across the 8-week period is summarized in Table 1. Exercise intensity was monitored based on the age-predicted maximum heart rate (HRmax) zones as specified in Table 1. Additionally, the Borg Rating of Perceived Exertion (RPE) was used to ensure that each participant maintained the targeted intensity zone, allowing for individual adjustments within the sessions. The progression was achieved by increasing the number of sets and incorporating more complex plyometric and resistance elements across the three training phases.

**Table 1:** The 8-week multi-component exercise training protocol.

Training Phase	Warm-up (5–10 min)	Main Exercise Phase (25–30 min)	Cool-down (5–10 min)	Target Intensity
**Weeks 1–2 (Adaptation)**	Joint mobility, light dynamic stretching	Focus on technique and bodyweight control. *Key exercises:* Y-T-W, hip bridge, plank, basic lunges, and airplane exercise. (2–3 sets)	Light static stretching, breathing	50-60% HRmax
**Weeks 3–5 (Progression)**	Dynamic stretching, light skipping	Introduction of dynamic and resistance elements. *Key exercises:* Rope skipping, T-push-up, sit-ups, plank with forward reach, and shoulder press. (3 sets)	Static stretching, flexibility	60-70% HRmax
**Weeks 6–8 (Optimization)**	Dynamic stretching, agility drills	Integration of power and plyometrics. *Key exercises:* Squat jumps, drop squats, medicine ball throw, and high-tempo rope skipping. (3–4 sets)	Deep static stretching	70-80% HRmax

Note: HRmax = Maximum Heart Rate (calculated as 220 – age).

### Data collection tools

Measurements were carried out in two stages: before (pre-test) and after (post-test) the 8-week exercise program.

- Balance: Flamingo Balance Test was used. Test-retest reliability is high [13].
- Flexibility: Sit-and-Reach Test was used. It offers high validity for hamstring extensibility [14].
- Aerobic capacity: 20-Meter Shuttle Run Test (Beep Test) was used to estimate VO_2_max [15].
- Agility: T-Test was used [16].
- Lower limb muscle strength: Vertical Jump Test was used [17].
- Speed: 20-Meter Speed Test was used [18].
- Anthropometric measurements: Height, body weight, and Body Mass Index (BMI) were measured. BMI is a key indicator associated with metabolic risk [19,20].

### Hematological measurements

Complete blood count (hemogram) was performed at pre-test and post-test stages. Blood samples were collected in the morning in a fasting state and evaluated using automated hematology analyzers (Sysmex XN-Series, Kobe, Japan). Parameters analyzed included hemoglobin (HGB), hematocrit (HCT), erythrocyte count (RBC), leukocyte count (WBC), platelet count (PLT), mean corpuscular volume (MCV), and mean corpuscular hemoglobin (MCH).

### Data analysis

Statistical analyses were performed using Jamovi 2.7.13 software. Data distribution was assessed using the Shapiro-Wilk test, visual inspection of Q-Q plots, and skewness and kurtosis coefficients [21,22]. Paired-samples t-tests were used to compare pre-test and post-test measurements of physical fitness and hematological parameters. Mean differences are reported with 95% confidence intervals. Effect sizes were calculated as Cohen’s dz for paired observations and interpreted using conventional thresholds of 0.2 (small), 0.5 (medium), and 0.8 (large). Exact p-values are reported whenever available; values smaller than 0.001 are reported as p < 0.001. Statistical significance was set at p < 0.05.

## Results

This section presents the pre-to-post comparisons observed during the 8-week exercise period. As shown in Table 2, statistically significant changes were observed in body composition and performance-related measures. Body weight and BMI decreased, whereas estimated VO_2_max, vertical jump, and flexibility increased. The times recorded for the 20 m sprint and agility test and the number of balance errors decreased, indicating better post-test performance. The effect sizes ranged from medium to large within the present sample.

**Table 2:** Comparison of pre-test and post-test physical fitness and anthropometric measures (N = 36).

	Measurement	Mean	SD	95% CI for mean difference	t	p	Cohen's dz (95% CI)
**Body Weight (kg)**	**Pre-test**	76.41	9.33	1.50, 2.18	11.02	<0.001*	1.84 (1.29, 2.37)
	**Post-test**	74.57	8.59				
**Body Mass Index (kg/m²)**	**Pre-test**	25.19	1.74	0.49, 0.70	12.02	<0.001*	2.00 (1.43, 2.57)
	**Post-test**	24.59	1.59				
**Estimated VO**_2_ **max (ml/kg/min)**	**Pre-test**	30.79	6.07	−1.90, −1.23	−9.54	<0.001*	1.59 (1.09, 2.08)
	**Post-test**	32.35	5.73				
**Vertical Jump (cm)**	**Pre-test**	31.89	13.45	−2.44, −1.83	−14.27	<0.001*	2.38 (1.73, 3.02)
	**Post-test**	34.03	13.65				
**Flexibility (cm)**	**Pre-test**	11.14	2.88	−0.48, −0.13	−3.49	<0.001*	0.58 (0.22, 0.93)
	**Post-test**	11.44	2.89				
**Balance (Error Count)**	**Pre-test**	1.58	1.30	0.79, 1.38	7.43	<0.001*	1.24 (0.80, 1.67)
	**Post-test**	0.50	0.77				
**20 m Speed (sec)**	**Pre-test**	4.29	1.27	0.03, 0.09	3.73	<0.001*	0.62 (0.26, 0.98)
	**Post-test**	4.23	1.28				
**Agility (sec)**	**Pre-test**	14.69	1.66	0.17, 0.34	5.80	<0.001*	0.97 (0.56, 1.36)
	**Post-test**	14.44	1.76				

***Note: The 95% CI for the mean difference refers to the paired difference calculated as pre-test minus post-test. Cohen’s dz is reported with its two-sided 95% confidence interval. Statistical significance was set at p < 0.05.***

As shown in Table 3, statistically significant pre-to-post increases were observed in HGB, HCT, RBC, WBC, and PLT values (all p < 0.001). No statistically significant changes were observed in MCV or MCH (p > 0.05). These findings describe changes in circulating hematological measures; the present data do not establish whether they reflect erythropoiesis, hemoconcentration, plasma-volume shifts, or other physiological variation.

**Table 3:** Comparison of pre-test and post-test hematological parameters (N = 36).

	Measurement	Mean	SD	95% CI for mean difference	t	p	Cohen's dz (95% CI)
**Hemoglobin (HGB) (g/dL)**	**Pre-test**	15.08	1.13	−0.73, −0.33	−5.32	<0.001*	0.89 (0.50, 1.27)
	**Post-test**	15.61	0.76				
**Hematocrit (HCT) (%)**	**Pre-test**	43.08	2.89	−1.31, −0.53	−4.73	<0.001*	0.79 (0.41, 1.16)
	**Post-test**	44.00	3.01				
**Erythrocyte (RBC) (10^6/μL)**	**Pre-test**	4.99	0.29	−0.29, −0.14	−5.78	<0.001*	0.96 (0.56, 1.36)
	**Post-test**	5.21	0.30				
**Leukocyte WBC (10^3/μL)**	**Pre-test**	6.43	0.94	−0.57, −0.38	−10.28	<0.001*	1.71 (1.19, 2.22)
	**Post-test**	6.91	0.86				
**Platelet (PLT) (10^3/μL)**	**Pre-test**	234.64	40.92	−51.62, −27.83	−6.78	<0.001*	1.13 (0.71, 1.54)
	**Post-test**	274.37	39.98				
**MCV (fL)**	**Pre-test**	84.79	4.02	−0.24, 0.03	−1.62	0.114	0.27 (−0.06, 0.60)
	**Post-test**	84.90	4.11				
**MCH (pg)**	**Pre-test**	29.49	1.90	−0.03, 0.17	0.35	0.726	0.06 (−0.27, 0.38)
	**Post-test**	29.47	1.86				

***Note: The 95% CI for the mean difference refers to the paired difference calculated as pre-test minus post-test. Cohen’s dz is reported with its two-sided 95% confidence interval. Statistical significance was set at p < 0.05.***

## Discussion

This exploratory study examined pre-to-post changes in physical fitness and selected hematological parameters during an 8-week multi-component exercise program in sedentary adults aged 30–45 years. The findings showed changes in body composition, estimated aerobic capacity, motor performance, and selected hematological measures. Because the study used a single-group pre-test/post-test design without a control group, the findings should be interpreted as preliminary within-participant observations rather than evidence of causal effects.

In the present sample, body weight and BMI decreased from pre-test to post-test. This pattern is consistent with previous studies reporting associations between regular physical activity and body composition [7,9]. Estimated VO_2_max and several motor-performance measures also changed in a favorable direction. These observations are compatible with previous evidence on structured exercise and physical fitness, but the design does not permit the changes to be attributed solely to the intervention [4].

Significant pre-to-post increases were observed in HGB, HCT, and RBC, whereas MCV and MCH remained statistically unchanged. This pattern describes quantitative changes in circulating hematological measures without detectable changes in the assessed erythrocyte indices. Although exercise has been associated with hematological responses in previous research [23–25], erythropoietin, hemoglobin mass, iron status, plasma volume, and hydration markers were not measured in this study. The observed increases therefore cannot be attributed to erythropoiesis or improved oxygen-carrying capacity and may partly reflect hemoconcentration, plasma-volume shifts, or other physiological variation.

Pre-to-post increases were also observed in WBC and PLT. Acute and chronic exercise can be accompanied by immune, inflammatory, and hemodynamic responses [26,27]; however, inflammatory markers, cytokines, platelet-activation measures, and serial post-exercise blood samples were not obtained. Consequently, the clinical or physiological meaning of these changes cannot be determined from the present data.

Several limitations should be acknowledged. First, the single-group design without a control group prevents the isolation of program-related changes from learning effects, natural physiological variation, regression to the mean, or unrecorded changes in diet, sleep, and physical activity. Causal conclusions therefore cannot be drawn. Second, the sample size was modest and limits generalizability. The sample included both women and men, but it was not large enough for adequately powered sex-stratified analyses; potential sex-related differences may therefore have influenced the findings. Third, although blood samples were collected in a standardized morning fasting state, dietary intake, supplement use, menstrual-cycle timing, and hydration status were not strictly monitored. Plasma volume, erythropoietin, hemoglobin mass, iron status, inflammatory markers, and platelet-activation markers were also not assessed. Accordingly, the observed changes in HGB, HCT, RBC, WBC, and PLT cannot be interpreted as evidence of erythropoietic adaptation, enhanced oxygen transport, or a beneficial immune or platelet response. Finally, estimated VO_2_max was derived from a field test rather than direct gas analysis.

## Conclusion

In conclusion, participation in the eight-week multi-component exercise program coincided with favorable pre-to-post changes in physical fitness and BMI and with measurable changes in selected hematological parameters in sedentary adults aged 30–45 years. HGB, HCT, RBC, WBC, and PLT increased, whereas MCV and MCH remained statistically unchanged. Because the study lacked a control group and did not assess plasma volume, hydration status, erythropoietin, hemoglobin mass, or iron status, the hematological changes cannot be assigned to a specific mechanism. Randomized controlled studies with larger samples, sex-stratified analyses, and more comprehensive physiological measurements are needed to confirm these observations.

## Supporting information

S1 Dataset Anonymized raw data. (XLSX)
